# Risk of Pneumonia with Inhaled Corticosteroid versus Long-Acting Bronchodilator Regimens in Chronic Obstructive Pulmonary Disease: A New-User Cohort Study

**DOI:** 10.1371/journal.pone.0097149

**Published:** 2014-05-30

**Authors:** Rachael L. DiSantostefano, Tim Sampson, Hoa Van Le, David Hinds, Kourtney J. Davis, Nawar Diar Bakerly

**Affiliations:** 1 Worldwide Epidemiology, GlaxoSmithKline, Research Triangle Park, North Carolina, United States of America; 2 Salford Royal NHS Foundation Trust and Manchester University, Manchester, United Kingdom; Maastricht University Medical Center (MUMC), Netherlands

## Abstract

**Introduction:**

Observational studies using case-control designs have showed an increased risk of pneumonia associated with inhaled corticosteroid (ICS)-containing medications in patients with chronic obstructive pulmonary disease (COPD). New-user observational cohort designs may minimize biases associated with previous case-control designs.

**Objective:**

To estimate the association between ICS and pneumonia among new users of ICS relative to inhaled long-acting bronchodilator (LABD) monotherapy.

**Methods:**

Pneumonia events in COPD patients ≥45 years old were compared among new users of ICS medications (n = 11,555; ICS, ICS/long-acting β_2_-agonist [LABA] combination) and inhaled LABD monotherapies (n = 6,492; LABA, long-acting muscarinic antagonists) using Cox proportional hazards models, with propensity scores to adjust for confounding. Setting: United Kingdom electronic medical records with linked hospitalization and mortality data (2002–2010). New users were censored at earliest of: pneumonia event, death, changing/discontinuing treatment, or end of follow-up. Outcomes: severe pneumonia (primary) and any pneumonia (secondary).

**Results:**

Following adjustment, new use of ICS-containing medications was associated with an increased risk of pneumonia hospitalization (n = 322 events; HR = 1.55, 95% CI: 1.14, 2.10) and any pneumonia (n = 702 events; HR = 1.49, 95% CI: 1.22, 1.83). Crude incidence rates of any pneumonia were 48.7 and 30.9 per 1000 person years among the ICS-containing and LABD cohorts, respectively. Excess risk of pneumonia with ICS was reduced when requiring ≥1 month or ≥ 6 months of new use. There was an apparent dose-related effect, with greater risk at higher daily doses of ICS. There was evidence of channeling bias, with more severe patients prescribed ICS, for which the analysis may not have completely adjusted.

**Conclusions:**

The results of this new-user cohort study are consistent with published findings; ICS were associated with a 20–50% increased risk of pneumonia in COPD, which reduced with exposure time. This risk must be weighed against the benefits when prescribing ICS to patients with COPD.

## Introduction

Pneumonia can result in significant morbidity and mortality, particularly among the elderly and patients with chronic obstructive pulmonary disease (COPD) [1]–[4]. Risk factors for the development of pneumonia, including pneumonia requiring hospitalization, have been well characterized in clinical and observational studies and include older age, current smoking status, low body mass index (BMI), chronic comorbid conditions (e.g., dementia, diabetes, cardiovascular disease), higher levels of dyspnea, and markers of COPD disease severity [5]–[8].

In patients with COPD, randomized controlled trials (RCT) [6], [9], meta-analyses [10]–[13] and observational studies [14]–[16] have generally observed an increased risk of pneumonia associated with the use of inhaled corticosteroid (ICS)-containing medications relative to non-steroid medications, including some evidence of a dose-related effect [10], [14], [16]. The mechanism by which ICS increase risk of pneumonia is unclear but may relate to reduced inflammatory response [17]. Comparisons across these individual studies have limitations, including disparate study populations and time periods, differing doses, molecules and devices, and variable definitions of pneumonia, which are discussed elsewhere [11].

Some previous observational studies that utilized a nested case-control design [14]–[16] have known disadvantages; most nested-case control designs combine prevalent and new users of ICS-containing medications, who may have different risks of pneumonia because of varying exposure time, and this may introduce a survivor or responder bias [18], [19]. In addition, these studies did not collect data on important risk factors for pneumonia, including lung function, smoking status, BMI, and clinically significant dyspnea. Examination of new medication users and collection of important confounding factors could offer advantages relative to past observational study designs to produce a less biased estimate of the association between ICS and pneumonia risk.

We aimed to improve upon the methods of prior observational studies and examine the association between ICS and pneumonia in new users of ICS-containing medications versus new users of long-acting bronchodilators (LABD) utilizing a general practice (GP), electronic-linked medical record database that included systematically collected COPD disease severity markers and other confounding factors. Preliminary results of these data have been published in abstract form [20].

## Methods

### Design

The source population included patients in the United Kingdom (UK) enrolled with a GP that contributes to the Clinical Practice Research Datalink GP OnLine Data database (CPRD GOLD, formerly referred to as General Practice Research Database [GPRD]) [21]. The CPRD GOLD database is representative of the age and gender distribution of the UK [22] and includes de-identified primary care electronic medical records containing demographic data, medical history, prescribed medications, diagnostic tests, specialist referrals, and secondary care information (e.g., hospitalization). COPD classification has previously been validated in an older version of CPRD-GOLD using the OXMIS coding system [23] and pneumonia hospital admissions have been validated more recently using READ codes and hospital identifiers in THIN, a similar UK electronic medical record [24]. This dataset is widely used in epidemiologic research, including in the study of COPD [2], [8], [21], [22], [25].Patients identified in the CPRD GOLD database were required to have both linked Hospital Episode Statistics (HES) [26] and vital statistics from Office for National Statistics [27]. Patients were required to have valid data in both CPRD and HES during the study period, including baseline and follow-up periods. HES data provides additional information about hospital admission not found in the primary care CPRD GOLD data, including primary and non-primary causes for each episode of in-patient care, type of admission (emergency versus non-emergency), length of stay and discharge status for approximately half of CPRD GOLD practices. The ONS data on death was considered the gold standard for mortality data in this study.

A new-user cohort design was used to examine the risk of pneumonia among patients initiating ICS-containing medication versus patients initiating LABD (long-acting beta_2_ agonists [LABA] or long-acting muscarinic antagonists [LAMA]) with a diagnosis of COPD in the year prior to the index prescription (including index date). Patients were ≥45 years old and free of pneumonia at the index date, with at least 1 year of baseline data without use of ICS or LABD prior to their index prescription. Patients with concurrent asthma were included; however, patients with a diagnosis that was not compatible with COPD (e.g., cystic fibrosis, pulmonary fibrosis, and bronchiectasis) were excluded. Planned feasibility analysis conducted prior to the study suggested that the proposed analysis period of 2005–2010 would not yield sufficient precision to detect meaningful differences between treatment groups and therefore the study period was expanded to 2002–2010 in order to ensure detection of hazards ratio of 1.85 or smaller with at least 80% power based on observed background rates of pneumonia in the new-user cohort and the number of severe events [28].

Based on the treatment paradigm for COPD in the Global Initiative for Chronic Obstructive Lung Disease strategy document, there are potential differences in COPD severity between new-users of ICS-containing medications and LABDs [29]. A LABD is recommended as initial maintenance treatment in patients with COPD with the addition of a second bronchodilator or ICS if disease severity warrants. Propensity scores (PS) were used to adjust for these potential differences. Additionally, patients receiving multiple LABDs (LABA+LAMA) or triple therapy (LABA + LAMA + ICS) were excluded to minimize confounding by severity.

Ethics approval was obtained from the Independent Scientific Advisory Committee (ISAC), which oversees research in CPRD (protocol 12_074R). This protocol is also registered with the European Network of Centres for Pharmacoepidemiology and Pharmacovigilance (ENCePP) (protocol reference ENCEPP/SDPP/4093).

### Outcomes

Pneumonia outcomes were recorded in either in primary care (CPRD GOLD) or HES. Pneumonia CPRD GOLD codes were based in part on those published by others [8], [24] and modified to include HES hospital codes (ICD-10) with feedback from a UK physician (NDB), an infectious disease physician (LM), a clinical consultant (CT), and the Independent Scientific Advisory Committee reviewer. The final set of pneumonia codes included 106 HES codes and 197 CPRD GOLD codes (Tables S1 and S2). Despite using an expanded list, the majority of pneumonia diagnoses in primary care or HES were limited to a few codes, i.e., the top four codes in each of HES and CPRD GOLD identified 95% and 80% of all pneumonias, respectively (Tables S3 and S4). Overall, all pneumonias were identified by 23 of the HES codes and 17 of the CPRD GOLD codes. The top three codes for both HES and CPRD GOLD were pneumonia “unspecified”, “not otherwise specified” and “unspecified organism”.

A pneumonia episode approach described elsewhere [8] was used. Briefly, all pneumonia events were defined by a start and end date, with most episodes lasting 70 days (10 weeks), irrespective of treatment duration. 70 days was considered sufficient for lung function and clinical conditions to have returned to baseline. Episodes started at either the earliest diagnosis of pneumonia or antibiotics prescription within 3 days prior to pneumonia diagnosis. Episodes ended when an individual had died, follow-up data ended, and/or was free of pneumonia diagnosis and antibiotics for at least 14 days following the 70-day interval. Our primary analysis focused on the first pneumonia event (severe and overall pneumonia events) following entry into the study; however, patients were eligible to experience a subsequent pneumonia 14 days following an episode end.

Our primary definition of pneumonia was severe pneumonia, defined as hospitalization for pneumonia or death (for any reason) during a pneumonia episode. The term chosen for our primary outcome, “severe pneumonia”, was chosen to avoid confusion with “serious pneumonia” used in clinical trials. A serious adverse event in clinical trials has a specific meaning and refers to events that require expedited reporting to regulators and that are life-threatening or that result in hospitalization or death. Infectious disease specialists also grade the severity of pneumonia by validated indices that relate to determining treatment strategies and/or risk of fatality, but pneumonia severity scores such as CURB-65 (confusion, uremia, rate respiratory, blood pressure, age >65) or PSI (pneumonia severity index) were not available [30].

Any pneumonia was examined as a secondary outcome. As 90% of pneumonia episodes resulted in hospitalization where pneumonia was listed as a primary or secondary cause during the hospitalization, additional post-hoc analyses to assess sensitivity were performed regarding the position of the pneumonia diagnosis across episodes of inpatient care. A gradient from more sensitive to more specific pneumonia definitions were therefore used in this study: (1) all pneumonia (secondary outcome); (2) severe pneumonia (primary outcome); (3) hospitalized pneumonia (pneumonia resulting in hospitalization where pneumonia was listed as the primary cause for any episode of inpatient care during a hospitalization); and (4) hospitalized with pneumonia on the first inpatient care episode (pneumonia was listed as the primary cause for the first episode of care during a hospitalization).

### Exposure

The primary exposure of interest was ICS-containing medications, while the comparator exposure group was LABD without ICS. Adherence to maintenance medications is poor among COPD patients with patients dispensed maintenance medications covering an average of less than half of a one-year time period [31]. Therefore, to determine the exposure period and account for poor adherence to respiratory medications, patients were classified as exposed to study medication for the duration of their treatment plus up to an additional 60-day grace period. Maintenance respiratory inhalers generally contain a 30-day supply of medication, and this grace period allowed 90 days between prescriptions, prior to censoring from the primary analysis. To identify a study population of persistent new users, we restricted to patients on treatment for ≥6 months. Our exposure period calculation methods and censoring following discontinuation is compatible with potential disease mechanisms such as immune suppression and have been used by others in COPD research [32].

To examine a potential dose-response relationship with ICS-containing medications, the strength of the prescribed ICS medication on the index prescription date was categorized based on classification in the Global Initiative for Asthma (GINA) guidelines [33] into low, medium, and high daily-dose of ICS (corresponding to estimated equipotent daily doses of beclomethasone dipropionate chlorofluorocarbon of 200–500 mcg, >500–1000 mcg and >1000–2000 mcg, respectively).

### Confounding factors

Confounding factors were selected based on clinical importance relating to disease severity and pneumonia risk (Table 1). The CPRD-GOLD primary care record was the primary source for confounders, and HES was used to characterize hospitalization in the baseline period. Most confounding factors were determined in the 1-year baseline period with the entire patient record used for comorbidity classification. In addition, COPD severity and dyspnea were identified during 1 year prior to and 3 months following index date, to be consistent with the 15-month period allowed by the Quality Outcomes Framework (QOF) for lung function and the Medical Research Council (MRC) dyspnea assessment [40].

**Table 1:** Summary of Confounding Variables, by Category, Measured Prior to Index Prescription and by which Propensity Scores were Adjusted.

Demographics	Respiratory disease severity (past year)	General health	Co-medications associated previously with CAP (past year)
Age	Asthma	Healthcare use (in past year)	Statins [34]
		- Number of emergency hospitalizations	
		- Number of non-emergency hospitalizations	
		- Number of general practice visits	
Gender	COPD severity	Charlson comorbidity chapters as defined by Khan et al [36] minus HIV (too infrequent) and respiratory disease (ever)	ACE-inhibitors [35]
	- Lung function less than FEV_1_ % predicted		
	- Moderate to severe dyspnea (Medical Research Council >2)		
	- Number of moderate and severe COPD exacerbations		
Calendar year of index prescription	Respiratory medications	Additional comorbidities associated	Immunosuppressants [37]
	- Short-acting beta-agonists	with pneumonia (ever)	
	- Oral corticosteroids	- Depression	
	- Theophyllines	- Anxiety	
	- Oxygen use	- GERD	
	- Nebulized therapies		
Smoking status		Pneumonia (past year)	GERD medications
Body mass index		Vaccinations	Benzodiazepines and non-
		- Influenza (past year)	benzodiazepine sedatives [38]
		- Pneumococcal (past 5 years)	
Socio-economic status			
- Overall Social Deprivation Scores for England [39]			
- Townsend Score			
- Pneumonia in the baseline period (CAP and severe CAP)			

ACE: angiotensin-converting-enzyme; CAP: community-acquired pneumonia; FEV_1_: forced expiratory volume in 1s; GERD: gastroesophageal reflux disease.

### Statistical analysis

Patients were described according to their COPD disease severity, demographic characteristics, and comorbidities. Patients were followed from the date of their first eligible prescription (cohort entry date) until the earliest of the following: pneumonia event; death; treatment end (up to 90-day gap between repeat prescriptions allowed for each inhaler); ICS initiation (signifying switch among LABD new users); or follow-up end (transfer to a new practice, practice stops participating, HES or CPRD data ends).

To adjust for confounding by severity due to differential prescribing by physicians according to the baseline characteristics of patients between ICS-containing medications and LABD, propensity scores (PS) were utilized. The PS was estimated to model the probability of a patient receiving ICS-containing medications compared with receiving LABD given a patient's observed set of baseline confounding factors. The logistic models used to calculate the PS included available confounding factors (Table 1). Confounding factors with counts of healthcare encounters and exacerbation were parameterized into categories prior to fitting the final models.

Analysis was performed using a Cox proportional hazard model for time to first pneumonia event, with adjustment for confounding using PS produced with stabilized inverse probability of treatment weights (IPTW) [41]–[43]. This approach is deemed more appropriate than PS matching when there may be effect measure modification [44]. PS matching and stratification (e.g., deciles, quintiles) were conducted as a sensitivity analysis [44]–[46]. Proportionality assumptions were met, based on graphical assessment of log(-log(S(t))) curves, and by adding interactions with time to the models.

To identify the effect of exposure time and potential for protopathic bias, the effect of ICS on pneumonia events was evaluated restricting to patients who were prescribed treatment for ≥1 month and ≥6 months (persistent use) separately using ‘lagged’ time intervals. Protopathic bias may occur when a new prescription of ICS is prescribed differentially relative to LABD to patient experiencing an exacerbation that was later identified as pneumonia, rather than the scenario of a pneumonia occurrence following the ICS prescription. As ICS are used to reduce risk of exacerbation, the 1-month period immediately following the new ICS prescription was excluded to evaluate protopathic bias (time 0 set to 31 days), an approach that has been used by others [47]. In the event that longer-term exposure may also affect the risk of pneumonia, the 6-month exposure period was examined (time 0 set to 181 days). For each analysis, PS were re-generated to ensure that individuals in each new-user group were clinically similar after restriction to the subgroups defined by duration of exposure and/or dose. For example, the PS for persistent users were regenerated using the end of the 6-month period as the analysis start.

Results for the expanded study period (2002–2010) are presented; results for the initial study period (2005–2010) are also provided for transparency when time trends were noted and when additional pneumonia events were identified during final programming.

All analyses were performed using SAS (version 9.1.3; Cary, NC).

## Results

**Figure 1:**
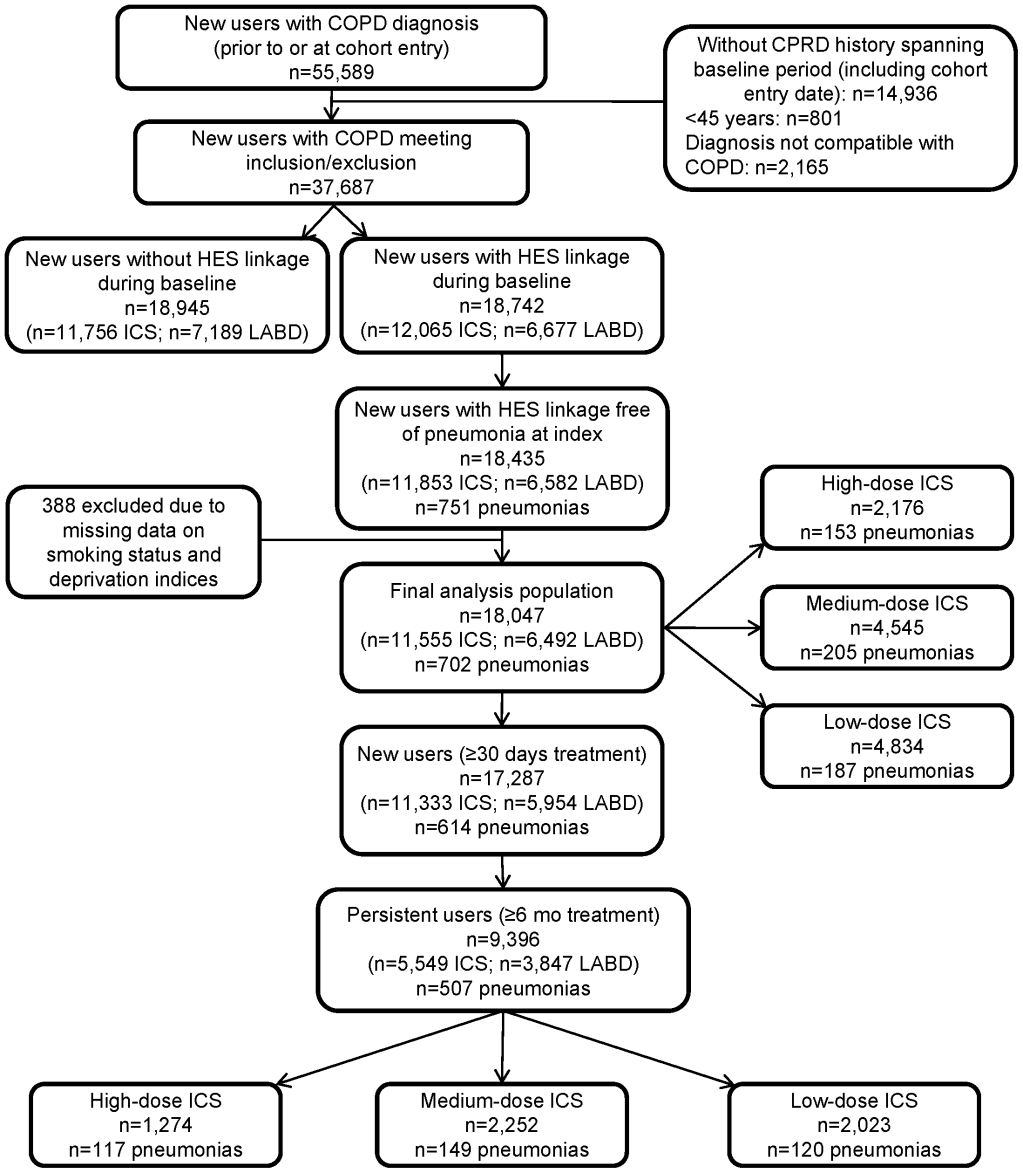
Patient record selection. n = 10,338,432 research-quality patients in CPRD-COPD; n = 645,287 new users of ICS-containing or new users of LABD **medications** COPD: chronic obstructive pulmonary disease; HES: Hospital Episode Statistics; ICS: inhaled corticosteroid; LABD: long-acting bronchodilator.

New users of ICS-containing or LABD medications (n = 645,287) were identified among CPRD GOLD patients between 2002–2010, of which 55,589 had a COPD diagnosis in the year prior to and/or at the index prescription. Figure 1 summarizes the number of patients fulfilling each of the required criteria. A total of 18,435 patients met all inclusion and exclusion criteria; 388 of these new users were excluded due to missing data on smoking status and deprivation indices resulting in a final analysis cohort of 18,047 new users at risk with 702 pneumonia events during follow-up.

Unadjusted incidence of any pneumonia was 48.7 and 30.9 per 1000 person years among the ICS-containing and LABD cohorts, respectively. Incidence of all pneumonia definitions by exposure, age, and sex before and after PS balancing are provided in Table S5. Mean time until censoring was approximately 1 year (353.4 days) and 9 months (285.8 days) among LABD and ICS-containing new users, respectively, with median times of approximately 5 months for both groups.

### Baseline characteristics

The ICS-containing cohort contained a higher proportion of non-smokers, unknown COPD severity, asthma diagnosis, and more emergency hospital admissions during baseline (Table 2). The LABD cohort had higher proportions of clinically significant dyspnea and ex-smokers, greater use of statins, ACE-inhibitors, and short-acting bronchodilators during baseline, and tended to have higher vaccination coverage. The two cohorts were similar regarding most comorbidities and current smoking status.

**Table 2:** Demographics from the Baseline Period (Year Before Cohort Entry) and Patient History for the Final Analysis Cohort Before and After Propensity Score Balancing.

Variable	Before propensity score balancing			After propensity score balancing (matched cohorts)		
	ICS-containing medications	LABD medications	p-value	ICS-containing medications	LABD medications	p-value
	N = 11,555	N = 6,492		N = 6,201	N = 6,201	
	n (%)	n (%)		n (%)	n (%)	
Male	6,332 (54.8)	3,778 (58.2)	<0.01	3,633 (58.6)	3,589 (57.9)	0.42
Age at cohort entry date, y						
45–64	3,835 (33.2)	1,938 (29.9)	<0.01	1,897 (30.6)	1,889 (30.5)	0.1
65–79	5,521 (47.8)	3,316 (51.1)		3,061 (49.4)	3,155 (50.9)	
≥80	2,199 (19.0)	1,238 (19.1)		1,243 (20.0)	1,157 (18.7)	
Smoking status prior to cohort entry date						
No	815 (7.1)	278 (4.3)	<0.01	283 (4.6)	278 (4.5)	0.96
Yes	5,160 (44.7)	2,899 (44.7)		2,750 (44.3)	2,763 (44.6)	
Ex-smoker	5,580 (48.3)	3,315 (51.1)		3,168 (51.1)	3,160 (51)	
COPD severity						
COPD diagnosis but spirometry conflicts	284 (2.5)	164 (2.5)	<0.01	154 (2.5)	163 (2.6)	1
FEV_1_% predicted <80%	761 (6.6)	485 (7.5)		475 (7.7)	469 (7.6)	
FEV_1_% predicted ≥80%	338 (2.9)	225 (3.5)		213 (3.4)	214 (3.5)	
FEV_1_ % predicted ≥50–<80%	1,862 (16.1)	1,537 (23.7)		1,381 (22.3)	1,382 (22.3)	
FEV_1_% predicted ≥30–<50%	1,277 (11.1)	915 (14.1)		883 (14.2)	864 (13.9)	
FEV_1_ % predicted <30%	253 (2.2)	170 (2.6)		163 (2.6)	167 (2.7)	
Unknown	6,780 (58.7)	2,996 (46.1)		2,932 (47.3)	2,942 (47.4)	
Moderate to severe dyspnea (Medical Research Council >2)	948 (8.2)	1,084 (16.7)	<0.01	865 (13.9)	894 (14.4)	0.46
Asthma diagnosis	2,186 (18.9)	776 (12.0)	<0.01	770 (12.4)	769 (12.4)	0.98
**Comorbidities**						
Pneumonia episode^1^	232 (2.0)	121 (1.9)	0.5	110 (1.8)	114 (1.8)	0.79
Influenza vaccination^1^	7,573 (65.5)	4,526 (69.7)	<0.01	4,321 (69.7)	4,284 (69.1)	0.47
Pneumococcal vaccination^2^	4,298 (37.2)	2,681 (41.3)	<0.01	2,565 (41.4)	2,532 (40.8)	0.55
BMI status						
No recording	1,115 (9.6)	476 (7.3)	<0.01	472 (7.6)	467 (7.5)	0.9
Underweight (<18.5)	574 (5.0)	356 (5.5)		343 (5.5)	341 (5.5)	
Low normal (18.5 to <21)	1,139 (9.9)	672 (10.4)		616 (9.9)	638 (10.3)	
High normal (21 to <25)	2,901 (25.1)	1,672 (25.8)		1,650 (26.6)	1,595 (25.7)	
Overweight (25 to <30)	3,452 (29.9)	1,891 (29.1)		1,797 (29.0)	1,813 (29.2)	
Obese (≥30)	2,374 (20.5)	1,425 (22.0)		1,323 (21.3)	1,347 (21.7)	
MI diagnosis	950 (8.2)	590 (9.1)	0.05	532 (8.6)	548 (8.8)	0.61
CHF diagnosis	959 (8.3)	568 (8.7)	0.3	540 (8.7)	531 (8.6)	0.77
Dementia diagnosis	95 (<1)	36 (<1)	0.04	39 (<1)	36 (<1)	0.73
GERD diagnosis or GERD prescription	5,467 (47.3)	3,247 (50.0)	<0.01	3,062 (49.4)	3,083 (49.7)	0.71
Peptic ulcer diagnosis	890 (7.7)	552 (8.5)	0.06	512 (8.3)	518 (8.4)	0.85
Peripheral vascular disease diagnosis	989 (8.6)	638 (9.8)	<0.01	598 (9.6)	590 (9.5)	0.81
Renal diseases diagnosis	876 (7.6)	703 (10.8)	<0.01	628 (10.1)	639 (10.3)	0.74
**Medication and healthcare utilization**						
Oral corticosteroids (>4 Rx)^1^	335 (2.9)	218 (3.4)	0.09	206 (3.3)	202 (3.3)	0.84
Oxygen^1^	189 (1.6)	84 (1.3)	0.07	90 (1.5)	84 (1.4)	0.65
Nebulized therapy^1^	396 (3.4)	162 (2.5)	<0.01	176 (2.8)	159 (2.6)	0.35
SABD^1^	8,066 (69.8)	4,738 (73.0)	<0.01	4,534 (73.1)	4,488 (72.4)	0.35
Theophylline^1^	279 (2.4)	126 (1.9)	0.04	134 (2.2)	126 (2.0)	0.62
ACE-inhibitors^1^	2,785 (24.1)	1,742 (26.8)	<0.01	1,654 (26.7)	1,643 (26.5)	0.82
Statins^1^	3,351 (29)	2,372 (36.5)	<0.01	2,185 (35.2)	2,207 (35.6)	0.68
Count of GP visits^1^						
0	159 (1.4)	43 (<1)	<0.01	66 (1.1)	43 (<1)	0.26
1–5	2,803 (24.3)	1,537 (23.7)		1,426 (23.0)	1,464 (23.6)	
6–10	3,703 (32.0)	2,108 (32.5)		1,986 (32.0)	2,012 (32.4)	
11–15	2,405 (20.8)	1,332 (20.5)		1,269 (20.5)	1,279 (20.6)	
16–20	1,205 (10.4)	741 (11.4)		729 (11.8)	705 (11.4)	
≥21	1,280 (11.1)	731 (11.3)		725 (11.7)	698 (11.3)	
Count of emergency hospital admissions^1^						
0	9,008 (78.0)	5,365 (82.6)	<0.01	5,098 (82.2)	5,100 (82.2)	0.98
1–2	2,297 (19.9)	1,028 (15.8)		1,003 (16.2)	1,004 (16.2)	
≥3	250 (2.2)	99 (1.5)		100 (1.6)	97 (1.6)	
Count of non-emergency hospital admissions^1^						
0	9,416 (81.5)	5,168 (79.6)	<0.01	4,956 (79.9)	4,950 (79.8)	0.61
1–2	1,887 (16.3)	1,174 (18.1)		1,117 (18)	1,107 (17.9)	
≥3	252 (2.2)	150 (2.3)		128 (2.1)	144 (2.3)	
Count of moderate COPD exacerbations^1^						
0	7,180 (62.1)	4,272 (65.8)	<0.01	4,032 (65)	4,052 (65.3)	0.93
1	3,063 (26.5)	1,598 (24.6)		1,562 (25.2)	1,545 (24.9)	
≥2	1,312 (11.4)	622 (9.6)		607 (9.8)	604 (9.7)	

1 Recorded in baseline period.

2 Recorded up to 5 years prior to cohort entry date.

ACE: angiotensin-converting-enzyme; CAP: community-acquired pneumonia; CHF: congestive heart failure; FEV_1_: forced expiratory volume in 1s; GERD: gastroesophageal reflux disease; GP: general practitioner; ICS: inhaled corticosteroid; LABD: long-acting bronchodilator; MI: myocardial infarction; Rx: prescription.

### Propensity score balancing

After PS balancing, confounding factors were similar across treatment groups (Table 2). PS overlap was sufficient and no patients were excluded due to an overly influential IPTW.

### ICS and pneumonia (primary model and sensitivity analyses)

**Figure 2:**
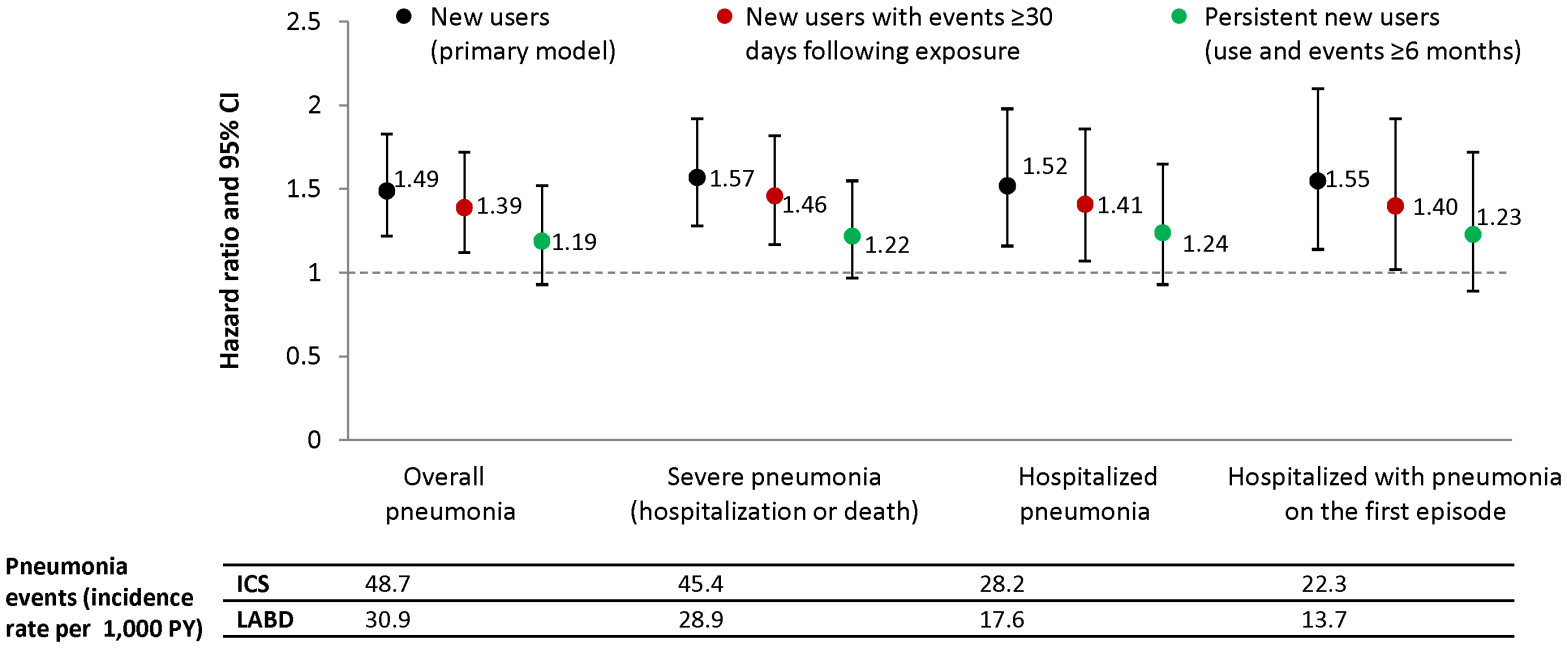
Pneumonia by different definitions among new users of ICS-containing and LABD medications (primary and sensitivity models). Data are hazard ratios and 95% CI for ICS compared with LABD. The incidence rates of pneumonia events per 1,000 person years for each group are presented below the figure for the primary model only. ICS: inhaled corticosteroid; LABD: long-acting bronchodilator.

Based on the primary IPTW analysis model of time to first pneumonia, new use of ICS-containing medications was associated with a statistically significantly increased risk of pneumonia relative to new use of LABD. This was observed for all pneumonia (hazard ratio [HR] = 1.49, 95% CI: 1.22, 1.83), severe pneumonia (HR = 1.57, 95% CI: 1.28, 1.92), hospitalized pneumonia (HR = 1.52, 95% CI: 1.16, 1.98), and hospitalized pneumonia with pneumonia as the primary cause on the first episode of care (HR = 1.55, 95% CI: 1.14, 2.10) (Figure 2, Table S6).

To address potential protopathic bias, patients with at least 30 days of therapy and events occurring after 30 days were analyzed. The increased risk of pneumonia remained significant; however, the magnitude was somewhat attenuated relative to the primary analysis (Figure 2, Table S6). When requiring persistent use of ICS, the HRs were further attenuated and no longer statistically significant (Figure 2, Table S6); about half of patients were censored prior to 6 months of use.

Results from 2005–2010 show a somewhat greater ICS treatment effect than the overall period, ranging in magnitude from 1.61 (95% CI: 1.29, 2.03) for the severe pneumonia outcome to 1.70 (95% CI: 1.27, 2.27) for the hospitalized pneumonia outcome (Table S6). This time trend potentially reflects changes in prescribing patterns over time, from low-dose ICS in earlier periods to medium- or high-dose fixed-dose combination therapy.

### ICS dose-response analysis

**Figure 3:**
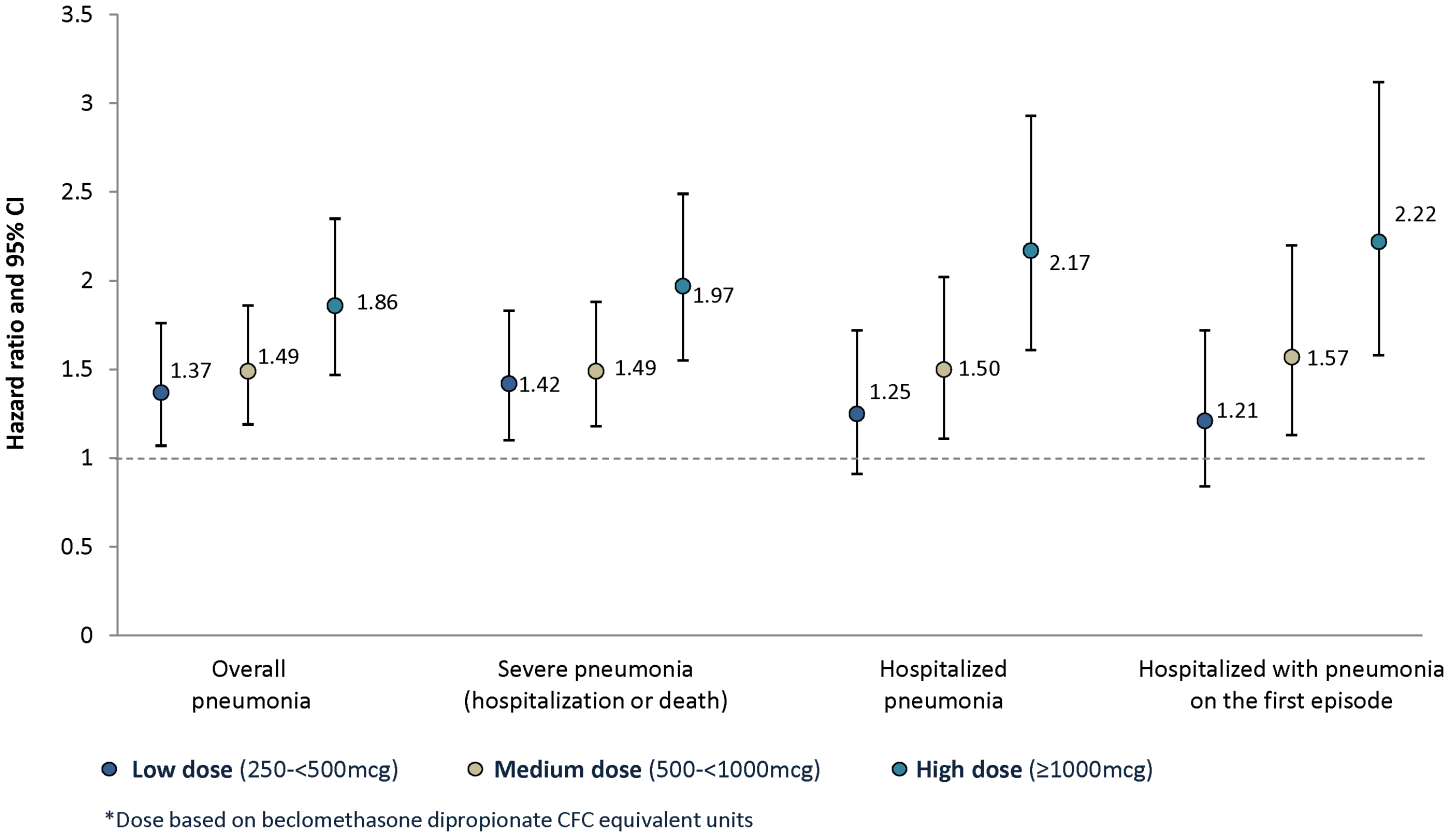
Pneumonia by different definitions and ICS daily dose in new users of ICS-containing medications. Data are hazard ratios and 95% CI for ICS compared with LABD. CFC: chlorofluorocarbon; ICS: inhaled corticosteroid; LABD: long-acting bronchodilator.

Increased hazard of pneumonia was observed in a potential dose-related trend for pneumonia outcomes, however, CIs were overlapping and residual confounding by severity cannot be ruled out as contributing to this trend (Figure 3, Table S6).

## Discussion

Evidence generated from this observational study is complementary to findings in RCTs. Crim et al. [6] noted an approximately 50% increase in the risk of pneumonia (HR = 1.52, 95% CI: 1.32, 1.76) among the fluticasone propionate (FP) treatment arms relative to placebo in a 3-year RCT. Our study reports that new use of ICS-containing medications was associated with an increased risk of pneumonia relative to LABD (HR = 1.49, 95% CI: 1.22, 1.83) in a population-based COPD cohort. Interestingly, our study suggests an attenuation of risk of pneumonia following persistent use of at least 6 months (HR = 1.19, 95% CI: 0.93, 1.52), whereas RCTs require about 6 months of use before treatment differences emerge. Meta-analyses of RCTs show attenuation of the risk of pneumonia after >2 years [10]. The difference between these results is not fully understood. The attenuation of risk with time in both observational and RCT settings may be related differential dropout of the most susceptible patients to pneumonia [48], [49].

Differences between the diagnosis of pneumonia in this observational study compared with clinical trials may help to interpret any differential results. The unadjusted incidences of pneumonia (per 1000 person years) in the present study for the ICS-containing and LABD cohorts, respectively (48.7 and 30.9) are lower than reported in the 3-year TORCH study of FP/salmeterol combination (84–88 and 52) [6] and in an analysis of two 1-year studies of fluticasone furoate/vilanterol (FF/VI) combination (79–95 and 42) [50]. These differences could be related to many factors including the rigor of ascertainment of pneumonia in the FF/VI studies and TORCH versus the present study, and how pneumonia was defined. Regarding this latter point, pneumonias in the present study would most likely have been defined as serious pneumonias in clinical trials [631 of 702 events (90%) were associated with inpatient admissions in the UK healthcare system], the unadjusted incidences of which are similar to TORCH (52–55 and 30) [6] and the 1-year FF/VI studies (35–43 and 12) [50]. The diagnosis of pneumonia in patients with COPD is imperfect and subject to misclassification. COPD patients may have chronically abnormal chest examinations and chest radiographs; and clinical signs and symptoms associated with pneumonia are similar to those associated with COPD exacerbation. In databases, pneumonia identification is even more challenging, as it is based upon existing information collected in routine healthcare that may not include all details measured in clinical trials (e.g, chest x-ray). Chest x-ray procedure codes and results are unavailable in the CPRD-GOLD database; therefore, we could not perform further analysis of our pneumonia definitions based on presence of chest x-ray results. Despite the challenges of accurate pneumonia identification, it is reassuring that the majority of pneumonia events in our study involved hospitalization, and therefore may have been more rigorously diagnosed than in a primary care setting where chest x-rays may not be ordered. It is possible that we have under-ascertainment of non-serious pneumonia events that may have been reported as an exacerbation in the electronic medical record and where chest x-rays would be less likely to be obtained.

Our definition of pneumonia was intended to balance sensitivity and specificity and excluded terms that may include exacerbations (e.g., lower respiratory tract infections), which have been shown to be reduced by ICS in COPD [51]. Interestingly, though our definition contained numerous codes (see Methods, Table S1 and S2), most were not used. Additionally, evidence suggests that ICS use may be associated with increased risk of other respiratory infections, such as non-tuberculosis mycobacterium [52] and tuberculosis, but not significantly associated with influenza [53]. Therefore, the terms used to define pneumonia and validation of recorded pneumonia diagnoses are important considerations in observational studies. Our hospitalized pneumonia codes included those that performed well in identifying acute hospitalized pneumonia episodes according to validation [24], and the differences in applied definitions may relate to some variation in effect measures observed across studies.

We noted channeling of more severe patients to ICS medications and although requiring 30 days of treatment or 6 months of treatment attenuated the observed HRs, pneumonia was still modestly associated with ICS exposure in persistent users. In addition, there were higher rates of pneumonia observed among patients prescribed the highest daily doses of ICS. Most of the prescribed high-dose ICS was fixed-dose combination (77.5%), whereas low dose was ICS monotherapy (80.6%), which could also reflect channeling of more severe patients to higher-dose ICS included in the fixed-dose combination products.

Other observational studies, which tended to be case-control designs, found a similar risk of pneumonia (Table S5) [14]–[16], [54], [55]. These studies used various approaches to adjust for confounding (e.g., limiting to newly diagnosed COPD, adjusting for OCS and ICS), but were often restricted to specific populations (e.g., veterans or Medicare patients) and were among older population (≥65 years old), along with tending to have shorter, earlier study periods compared with this study. Therefore our findings may have broader, or at least different, generalizability for the population of interest based on the use of a general population cohort, over a long study period, among a broader age group (≥45 years old). Two recent observational studies suggest a lower risk of pneumonia with budesonide than FP; however, the extent to which confounding by severity has been adjusted is unclear from the information given [55], [56]. In the observational study conducted in Sweden, the majority of fixed-dose combination medications prescribed were budesonide/formoterol (∼72%) [56] and the authors compared FP at a high daily dose [33] of 783 ± 338 mcg/day to budesonide at a medium daily dose [33] of 568 ± 235 mcg/day, without limiting the analysis to equipotent doses and similar time periods. While the Canadian study [55] was conducted over an 18-year study period (1990–2007) that occurred when most ICS prescribing was off-label, with no analytical accounting for substantial changes in available treatments and the evolving role of ICS and ICS/LABA in COPD treatment guidelines. During the study period, ICS monotherapy was not approved for use in COPD, and only one fixed-dose combination was approved during the study period (FP/salmeterol in 2003) [55]. It was not possible to evaluate differences in the risk of pneumonia within the ICS class in our analysis due to small numbers of patients using the two available fixed-dose combinations at equipotent doses and matching time periods. RCTs [6], [9], [51] and meta-analyses [11], [13] do not suggest intra-class differences; however, head-to-head randomized controlled trials are required to robustly evaluate this question.

We attempted to improve upon the limitations of prior observational work with a new-user design, which starts patient follow-up with the initial medication prescription. The design avoids potential biases from examining prevalent users relating to survivor bias and changes in covariates based on exposure to treatment [19]. However, the design may reduce sample size and precision relative to alternative designs that include prevalent and incident users. Additionally, we used a data source that captures important confounding factors and measures of disease severity to create a more robust PS to adjust for channeling bias (e.g., BMI, lung function, smoking, clinically significant dyspnea). Our methods are most similar to those conducted by Ernst [14] and Suissa [55], as the case-control study using incidence density sampling of new users would be mathematically equivalent to our Cox model provided the incidence rate is constant over time [57]. However, the differing time periods, pneumonia definitions, and allowance of other respiratory medications make direct comparison difficult.

Time trends noted in this study require further evaluation. As a result of a feasibility assessment prior to the conduct of the study, the study period was expanded from 2005–2010 to 2002–2010 to improve precision. At the beginning of the expanded period, ICS monotherapy was recommended as an initial treatment for COPD and was used at low doses. In May 2003, ICS/LABA was indicated in the UK for COPD patients with <50% predicted FEV_1_, and in May 2007 the indication was expanded to COPD patients with<60% predicted FEV_1_. Additionally, around 2005, spirometry became a reimbursable activity in the UK as part of the QOF, potentially affecting the number of COPD diagnoses, as well as the granularity recorded in CPRD GOLD on residual confounding factors. Upon the approval of ICS/LABA combinations in 2003 (fluticasone propionate/salmeterol) higher doses of ICS were prescribed. Differential risk over the study period was also observed, including an increased risk of pneumonia from 2005 onwards versus earlier time periods (data not shown), but whether this finding relates to a shift towards use of higher doses and fixed-dose combinations requires further evaluation.

Known limitations of non-randomized database analyses of comparative medication safety include the potential for confounding by severity when comparing users to non-users of a drug class, or when comparing medicines within a class in the setting of differing approved daily dose equivalents, labeled indications or market share. There is some evidence that patients prescribed ICS-containing medications in our study and higher doses thereof may have had more severe COPD, more frequent historical mention of comorbid asthma, and a higher risk of exacerbation than patients who received LABD without ICS. Residual confounding, particularly for patients with missing lung function data prior to QOF, or the lack of health status measures like St. George's Respiratory Questionnaire or COPD Assessment Test in the electronic medical record, may not have been fully adjusted for in the analysis.

Despite the limitations, this study provides important insights into the risk of pneumonia in a real-world setting among new users of ICS-containing and LABD medications prescribed for COPD. The increased risk of pneumonia observed in this study, which was not sensitive to varying pneumonia definitions, is consistent with RCTs and previous observational studies. Due to differences in the methods, as well as study population and timeframe, the risk of pneumonia we observed may be more broadly and reliably generalized to the COPD population, but physicians must weigh the risk of pneumonia relative to the benefits of ICS in COPD in light of each patient's profile and the goal of reducing risk of future exacerbations and maximizing health status. To improve understanding and aid physician decision making, future studies will ideally focus on further quantifying both benefits and risks in well-characterized subgroups of patients to help determine who is best treated with ICS-containing regimens, at what stage of the disease, at what dose, and how to monitor risk as part of disease management alongside smoking cessation, vaccinations, treatment of comorbidities, exercise and rehabilitation programs. Trade-offs between the improved control of confounding with randomized trial designs versus the generalizability and power of real-world observational studies is important to consider when designing and interpreting studies for specific questions of comparative effectiveness and safety. Rigorous and regular review of the totality of the evidence will continue to advance our understanding and better target treatment options to improve outcomes for COPD patients.

## Supporting Information

Table S1 ICD-10 pneumonia code used to identify pneumonia events. *Descriptions were taken directly from the ICD-10.
(DOCX)

Table S2 CPRD GOLD medcodes used to identify pneumonia. [X] Denotes the working diagnosis as recorded by the general practitioner. *Descriptions were taken directly from the CPRD-COPD.
(DOCX)

Table S3 ICD-10 pneumonia code recorded among 751 patients with pneumonia in new user cohort. 1. Patients may have multiple recordings of pneumonia codes. 2. HES ICD-10 codes recorded. *Descriptions were taken directly from the ICD-10.
(DOCX)

Table S4 CPRD GOLD medcodes recorded among 751 patients with pneumonia. 1. Note that patients may have multiple recordings of pneumonia codes. 2. GPRD Medcodes recorded. *Descriptions were taken directly from the CPRD-COPD.
(DOCX)

Table S5 Incidence of first pneumonia per 1000 person years for the final analysis cohort before and after propensity score balancing. IR: Incidence rate; PY: person years; Y: years. Severe pneumonia- pneumonia episode due to hospitalization or death during the pneumonia episode not censoring for prior non-severe pneumonia episode(s).
(DOCX)

Table S6 Hazard ratio and 95% CI of the association between first pneumonia and initiating medication use: applying sensitive to specific pneumonia definitions. CI: confidence interval; HR: hazard ratio; ICS: inhaled corticosteroids; LABD: long-acting bronchodilator. 1. Patients with a severe pneumonia episode due to a HES episode (hospitalization) with pneumonia as a primary diagnosis during an episode of care within the HES episode and not censored for other reasons during the severe pneumonia episode before the primary diagnosis. 2. Patients with a severe pneumonia episode due to hospitalization or death during the pneumonia episode not censoring for prior non-severe pneumonia episode(s). 3. Inverse probability of treatment weights method.
(DOCX)

Table S7 Summary of studies investigating the association between pneumonia and use or non-use of ICS. ACE: angiotensin-converting-enzyme; CAP: community-acquired pneumonia; CHF: congestive heart failure; ER: emergency room; FEV_1_: forced expiratory volume in 1s; GERD: gastroesophageal reflux disease; GP: general practitioner; HR: hazard ratio; ICS: inhaled corticosteroid; LABA: long-acting β_2_-agonist; LABD: long-acting bronchodilator; MI: myocardial infarction; NSAIDS: non-steroidal anti-inflammatory drug; OCS  =  oral corticosteroid; OR: odds ratio; RR: rate ratio; Rx: prescription; SABD: short-acting bronchodilator; VA: Veterans Affairs.
(DOCX)
