# Supplementary material for: Risk of Pneumonia with Inhaled Corticosteroid versus Long-Acting Bronchodilator Regimens in Chronic Obstructive Pulmonary Disease: A New-User Cohort Study
Source: PLoS One. 2014 May 30;9(5):e97149. doi: 10.1371/journal.pone.0097149 (PMC4039434; doi:10.1371/journal.pone.0097149)
Supplement: Table S1 — ICD-10 pneumonia code used to identify pneumonia events. *Descriptions were taken directly from the ICD-10. (DOCX) [file pone.0097149.s001.docx]

Table S1. ICD-10 pneumonia code used to identify pneumonia events

| **ICD-10 diagnosis code** | **Description*** |
| --- | --- |
| J69 | Pneumonitis due to solids and liquids |
| J690 | Pneumonitis due to food and vomit |
| J691 | Pneumonitis due to oils and essences |
| J698 | Pneumonitis due to other solids and liquids |
| O740 | Asp pneumonitis due to anaesthesia during labour and deliv |
| B671 | Echinococcus granulosus infection of lung |
| J173 | Pneumonia in parasitic diseases |
| J16 | Pneumonia due to other infectious organisms NEC |
| J168 | Pneumonia due to other specified infectious organisms |
| J17 | Pneumonia in diseases classified elsewhere |
| J178 | Pneumonia in other diseases classified elsewhere |
| J18 | Pneumoniaorganism unspecified |
| J180 | Bronchopneumonia, unspecified |
| J181 | Lobar pneumonia, unspecified |
| J188 | Other pneumonia, organism unspecified |
| J189 | Pneumonia, unspecified |
| A065 | Amoebic lung abscess |
| J85 | Abscess of lung and mediastinum |
| J850 | Gangrene and necrosis of lung |
| J851 | Abscess of lung with pneumonia |
| J852 | Abscess of lung without pneumonia |
| B206 | HIV disease resulting in Pneumocystis carinii pneumonia |
| B371 | Pulmonary candidiasis |
| B380 | Acute pulmonary coccidioidomycosis |
| B381 | Chronic pulmonary coccidioidomycosis |
| B382 | Pulmonary coccidioidomycosis, unspecified |
| B390 | Acute pulmonary histoplasmosis capsulati |
| B392 | Pulmonary histoplasmosis capsulati, unspecified |
| B400 | Acute pulmonary blastomycosis |
| B402 | Pulmonary blastomycosis, unspecified |
| B410 | Pulmonary paracoccidioidomycosis |
| B420 | Pulmonary sporotrichosis |
| B450 | Pulmonary cryptococcosis |
| B460 | Pulmonary mucormycosis |
| B583 | Pulmonary toxoplasmosis |
| B59 | Pneumocystosis |
| B590 | Pneumocystosis |
| B59X | Pneumocystosis |
| J172 | Pneumonia in mycoses |
| A15 | Respiratory TB bacteriologically and histologically confirmed |
| A150 | TB lung confirm sputum microscopy with or without culture |
| A151 | Tuberculosis of lung, confirmed by culture only |
| A152 | Tuberculosis of lung, confirmed histologically |
| A153 | Tuberculosis of lung, confirmed by unspecified means |
| A154 | TB intrathoracic lymph nodes confirm bact histologically |
| A155 | Tuberculosis of larynx, trachea & bronchus conf bact/hist'y |
| A156 | Tuberculous pleurisy, conf bacteriologically/his'y |
| A157 | Primary respiratory TB confirm bact and histologically |
| A158 | Other respiratory TB confirm bact and histologically |
| A159 | Respiratory TB unspec confirm bact and histologically |
| A16 | Respiratory TB not confirmed bacteriologically or histologically |
| A160 | Tuberculosis of lung, bacteriologically & histolog'y neg |
| A161 | Tuberculosis lung bact and histological examin not done |
| A162 | TB lung without mention of bact or histological confirm |
| A163 | TB intrathoracic lymph node without bact or hist confirm |
| A164 | TB larynx trachea and bronchus without bact or hist confirm |
| A165 | TB pleurisy without mention of bact or histological confirm |
| A167 | Prim respiratory TB without mention of bact or hist confirm |
| A168 | Oth respiratory TB without mention of bact or hist confirm |
| A169 | Resp TB unspec without mention of bact or hist confirm |
| A19 | Miliary tuberculosis |
| A190 | Acute miliary tuberculosis of a single specified site |
| A191 | Acute miliary tuberculosis of multiple sites |
| A192 | Acute miliary tuberculosis, unspecified |
| A198 | Other miliary tuberculosis |
| A199 | Miliary tuberculosis, unspecified |
| A310 | Pulmonary mycobacterial infection |
| J65 | Pneumoconiosis associated with tuberculosis |
| J650 | Pneumoconiosis associated with tuberculosis |
| J65X | Pneumoconiosis associated with tuberculosis |
| B012 | Varicella pneumonia |
| B052 | Measles complicated by pneumonia |
| J100 | Influenza with pneumonia, influenza virus identified |
| J110 | Influenza with pneumonia, virus not identified |
| J12 | Viral pneumonia, not elsewhere classified |
| J120 | Adenoviral pneumonia |
| J121 | Respiratory syncytial virus pneumonia |
| J122 | Parainfluenza virus pneumonia |
| J128 | Other viral pneumonia |
| J129 | Viral pneumonia, unspecified |
| J171 | Pneumonia in viral diseases classified elsewhere |
| A202 | Pneumonic plague |
| A212 | Pulmonary tularaemia |
| A221 | Pulmonary anthrax |
| A420 | Pulmonary actinomycosis |
| A430 | Pulmonary nocardiosis |
| A481 | Legionnaires' disease |
| J13 | Pneumonia due to Streptococcus pneumoniae |
| J130 | Pneumonia due to Streptococcus pneumoniae |
| J13X | Pneumonia due to Streptococcus pneumoniae |
| J14 | Pneumonia due to Haemophilus influenzae |
| J140 | Pneumonia due to Haemophilus influenzae |
| J14X | Pneumonia due to Haemophilus influenzae |
| J15 | Bacterial pneumonianot elsewhere classified |
| J150 | Pneumonia due to Klebsiella pneumoniae |
| J151 | Pneumonia due to Pseudomonas |
| J152 | Pneumonia due to staphylococcus |
| J153 | Pneumonia due to streptococcus, group B |
| J154 | Pneumonia due to other streptococci |
| J155 | Pneumonia due to Escherichia coli |
| J156 | Pneumonia due to other aerobic Gram-negative bacteria |
| J157 | Pneumonia due to Mycoplasma pneumoniae |
| J158 | Other bacterial pneumonia |
| J159 | Bacterial pneumonia, unspecified |
| J160 | Chlamydial pneumonia |
| J170 | Pneumonia in bacterial diseases classified elsewhere |

*Descriptions were taken directly from the ICD-10
