# Supplementary material for: Risk of Pneumonia with Inhaled Corticosteroid versus Long-Acting Bronchodilator Regimens in Chronic Obstructive Pulmonary Disease: A New-User Cohort Study
Source: PLoS One. 2014 May 30;9(5):e97149. doi: 10.1371/journal.pone.0097149 (PMC4039434; doi:10.1371/journal.pone.0097149)
Supplement: Table S2 — CPRD GOLD medcodes used to identify pneumonia. [X] Denotes the working diagnosis as recorded by the general practitioner. *Descriptions were taken directly from the CPRD-COPD. (DOCX) [file pone.0097149.s002.docx]

Table S2. CPRD GOLD medcodes used to identify pneumonia

| **GPRD Medical Code** | **Description*** |
| --- | --- |
| 9711 | Pneumonitis due to inhalation of solids or liquids |
| 10992 | Aspiration pneumonitis |
| 3847 | Pneumonitis due to inhalation of food or vomitus |
| 101204 | Aspiration pneumonia |
| 41781 | Pneumonitis due to inhalation of regurgitated food |
| 59083 | Pneumonitis due to inhalation of gastric secretions |
| 66104 | Pneumonitis due to inhalation of milk |
| 30996 | Milk inhalation pneumonitis |
| 45948 | Pneumonitis due to inhalation of vomitus |
| 56385 | Vomit inhalation pneumonitis |
| 25054 | Aspiration pneumonia due to vomit |
| 33837 | Pneumonitis due to inhalation of food or vomitus NOS |
| 56647 | Pneumonitis due to inhalation of oil or essence |
| 41015 | Lipoid pneumonia (exogenous) |
| 66773 | Pneumonitis due to inhalation of oil or essence NOS |
| 50876 | Asp pneumonitis due to anaesthesia during labour and deliv |
| 47504 | Pneumonitis due to inhalation of other solid or liquid |
| 54252 | Pneumonitis due to inhalation of solid or liquid NOS |
| 46066 | Pneumonitis due to inhalation of solid or liquid NOS |
| 99232 | [X]Pneumonitis due to inhalation of other solids and liquids |
| 13563 | Other aspiration pneumonia as a complication of care |
| 50408 | Ornithosis with pneumonia |
| 62408 | Lung fluke disease |
| 19992 | Lung echinococcus granulosus |
| 62623 | Pneumonia with ornithosis |
| 98782 | Pneumonia with toxoplasmosis |
| 10086 | Pneumonia and influenza |
| 25694 | Pneumonia due to other specified organisms |
| 30653 | Chest infection - pneumonia organism OS |
| 34251 | Pneumonia due to specified organism NOS |
| 40498 | Pneumonia with infectious diseases EC |
| 69782 | Pneumonia with other infectious diseases EC |
| 70559 | Pneumonia with other infectious diseases EC NOS |
| 66362 | Pneumonia with infectious diseases EC NOS |
| 886 | Bronchopneumonia due to unspecified organism |
| 16287 | Chest infection - unspecified bronchopneumonia |
| 572 | Pneumonia due to unspecified organism |
| 19400 | Chest infection - pneumonia due to unspecified organism |
| 9639 | Lobar pneumonia due to unspecified organism |
| 3683 | Basal pneumonia due to unspecified organism |
| 5324 | Atypical pneumonia |
| 11849 | Other specified pneumonia or influenza |
| 6094 | Pneumonia or influenza NOS |
| 98381 | [X]Pneumonia due to other specified infectious organisms |
| 53753 | [X]Other pneumonia, organism unspecified |
| 34732 | Amoebic lung abscess |
| 21185 | Abscess of lung and mediastinum |
| 29005 | Abscess of lung |
| 33730 | Single lung abscess |
| 37711 | Multiple lung abscess |
| 57667 | Gangrenous pneumonia |
| 35189 | Abscess of lung with pneumonia |
| 11202 | Abscess of lung NOS |
| 34659 | Abscess of lung and mediastinum NOS |
| 27641 | HIV disease resulting in Pneumocystis carinii pneumonia |
| 48481 | Candidiasis of lung |
| 40299 | Pneumonia - candidal |
| 54540 | Primary pulmonary coccidioidomycosis |
| 101507 | Histoplasma capsulatum with pneumonia |
| 91481 | Acute pulmonary histoplasmosis capsulati |
| 54551 | Chronic pulmonary histoplasmosis capsulati |
| 101292 | Histoplasma duboisii with pneumonia |
| 59951 | Pulmonary histoplasmosis |
| 41404 | Primary pulmonary blastomycosis |
| 100742 | Allergic bronchopulmonary aspergillosis |
| 54906 | Pulmonary cryptococcosis |
| 35220 | Pneumocystosis |
| 96332 | [X]Other pulmonary aspergillosis |
| 34274 | Pneumonia with aspergillosis |
| 52071 | Pneumonia with candidiasis |
| 103404 | Pneumonia with coccidioidomycosis |
| 53969 | Pneumonia with systemic mycosis NOS |
| 27519 | Pneumonia with pneumocystis carinii |
| 22011 | Primary tuberculous infection |
| 16265 | Primary tuberculous complex |
| 46272 | Tuberculous pleurisy in primary progressive tuberculosis |
| 42630 | Other primary progressive tuberculosis |
| 37694 | Primary tuberculous infection NOS |
| 635 | Pulmonary tuberculosis |
| 47336 | Lung tuberculosis |
| 53701 | Infiltrative lung tuberculosis |
| 48580 | Nodular lung tuberculosis |
| 16331 | Tuberculosis of lung with cavitation |
| 62468 | Tuberculosis of bronchus |
| 16741 | Tuberculous fibrosis of lung |
| 15693 | Tuberculous bronchiectasis |
| 9953 | Tuberculous pneumonia |
| 66441 | Tuberculous pneumothorax |
| 18950 | Other specified pulmonary tuberculosis |
| 38110 | Pulmonary tuberculosis NOS |
| 63959 | Other respiratory tuberculosis |
| 23472 | Tuberculous pleurisy |
| 37834 | Tuberculosis of pleura |
| 39512 | Tuberculous empyema |
| 14913 | Tuberculous hydrothorax |
| 56890 | Tuberculous pleurisy NOS |
| 58827 | Tuberculosis of intrathoracic lymph nodes |
| 5145 | Tuberculosis of hilar lymph nodes |
| 44129 | Tuberculosis of mediastinal lymph nodes |
| 49503 | Tuberculosis of tracheobronchial lymph nodes |
| 46926 | Tuberculosis of intrathoracic lymph nodes NOS |
| 69260 | Isolated tracheal or bronchial tuberculosis |
| 93015 | Isolated tracheal tuberculosis |
| 93948 | Isolated bronchial tuberculosis |
| 53473 | Isolated tracheal or bronchial tuberculosis NOS |
| 20333 | Tuberculous laryngitis |
| 31670 | Resp TB bacteriologically and histologically confirmed |
| 24413 | TB lung confirm sputum microscopy with or without culture |
| 93071 | Tuberculosis of lung, confirmed by culture only |
| 62530 | Tuberculosis of lung, confirmed histologically |
| 58588 | Tuberculosis of lung, confirmed by unspecified means |
| 44655 | TB intrathoracic lymph nodes confirm bact histologically |
| 44039 | Tuberculosis of larynx, trachea & bronchus conf bact/hist'y |
| 35443 | Tuberculous pleurisy, conf bacteriologically/histologically |
| 24517 | Primary respiratory TB confirm bact and histologically |
| 7133 | Respiratory TB not confirmed bact or histologically |
| 47832 | Tuberculosis of lung, bacteriologically & histolog'y neg |
| 41051 | Tuberculosis lung bact and histological examin not done |
| 40605 | Prim respiratory TB without mention of bact or hist confirm |
| 69471 | Resp TB unspcf,w'out mention/bacterial or histol confrmtn |
| 50902 | Other specified respiratory tuberculosis |
| 37598 | Tuberculosis of mediastinum |
| 72402 | Tuberculosis of nasopharynx |
| 97658 | Tuberculosis of nasal septum |
| 45861 | Tuberculosis of nasal sinus |
| 50147 | Other specified respiratory tuberculosis NOS |
| 16414 | Miliary tuberculosis |
| 72008 | Acute miliary tuberculosis |
| 31844 | Acute miliary tuberculosis of a single specified site |
| 42479 | Acute miliary tuberculosis of multiple sites |
| 32459 | Other specified miliary tuberculosis |
| 53331 | Miliary tuberculosis NOS |
| 32223 | Pulmonary mycobacterial infection |
| 24425 | Pulmonary mycobacterium avium-intracellulare infection |
| 73185 | [X]Other resp tubercul,confirmd bacteriologicly+histologically |
| 73225 | [X]Resp tuberculos unspcfd,confirmd bacteriolog+histologically |
| 55298 | [X]Resp TB unspcf, without mention/bacterial or histol confirmation |
| 97922 | [X]Miliary tuberculosis, unspecified |
| 63172 | Pneumoconiosis associated with tuberculosis |
| 47973 | Herpes simplex pneumonia |
| 32172 | Postmeasles pneumonia |
| 5202 | Viral pneumonia |
| 9389 | Chest infection - viral pneumonia |
| 67836 | Pneumonia due to adenovirus |
| 31269 | Pneumonia due to respiratory syncytial virus |
| 36675 | Pneumonia due to parainfluenza virus |
| 33478 | Viral pneumonia NEC |
| 14976 | Viral pneumonia NOS |
| 41034 | Pneumonia with measles |
| 43286 | Pneumonia with cytomegalic inclusion disease |
| 23726 | Pneumonia with varicella |
| 15912 | Influenza with pneumonia |
| 29457 | Chest infection - influenza with pneumonia |
| 13573 | Influenza with bronchopneumonia |
| 62632 | Influenza with pneumonia, influenza virus identified |
| 35745 | Influenza with pneumonia NOS |
| 52520 | [X]Other viral pneumonia |
| 53947 | [X]Pneumonia in viral diseases classified elsewhere |
| 58896 | Salmonella pneumonia |
| 70710 | Primary pneumonic plague |
| 47295 | Pneumonic plague, unspecified |
| 45161 | Pulmonary anthrax |
| 41084 | Wool-sorters' disease |
| 64306 | Pulmonary actinomycosis |
| 73340 | Pulmonary nocardiosis |
| 15308 | Legionella |
| 1849 | Lobar (pneumococcal) pneumonia |
| 29166 | Chest infection - pneumococcal pneumonia |
| 28634 | Other bacterial pneumonia |
| 22795 | Chest infection - other bacterial pneumonia |
| 23546 | Pneumonia due to klebsiella pneumoniae |
| 30591 | Pneumonia due to pseudomonas |
| 37881 | Pneumonia due to haemophilus influenzae |
| 48804 | Pneumonia due to haemophilus influenzae |
| 12423 | Pneumonia due to streptococcus |
| 63858 | Pneumonia due to streptococcus, group B |
| 5612 | Pneumonia due to staphylococcus |
| 50867 | Pneumonia due to other specified bacteria |
| 65419 | Pneumonia due to escherichia coli |
| 60299 | E. coli pneumonia |
| 45425 | Pneumonia due to proteus |
| 12061 | Pneumonia - Legionella |
| 52384 | Pneumonia due to other aerobic gram-negative bacteria |
| 43884 | Pneumonia due to bacteria NOS |
| 23095 | Bacterial pneumonia NOS |
| 60119 | Pneumonia due to Eaton's agent |
| 1576 | Pneumonia due to mycoplasma pneumoniae |
| 73735 | Pneumonia due to pleuropneumonia like organisms |
| 17025 | Chlamydial pneumonia |
| 30437 | Pneumonia with whooping cough |
| 35082 | Pneumonia with pertussis |
| 61623 | Pneumonia with actinomycosis |
| 67901 | Pneumonia with nocardiasis |
| 60482 | Pneumonia with Q-fever |
| 72182 | Pneumonia with salmonellosis |
| 49398 | Pneumonia with typhoid fever |
| 63763 | [X]Other bacterial pneumonia |

[X] Denotes the working diagnosis as recorded by the general practitioner

*Descriptions were taken directly from the CPRD-COPD
