# Supplementary material for: Risk of Pneumonia with Inhaled Corticosteroid versus Long-Acting Bronchodilator Regimens in Chronic Obstructive Pulmonary Disease: A New-User Cohort Study
Source: PLoS One. 2014 May 30;9(5):e97149. doi: 10.1371/journal.pone.0097149 (PMC4039434; doi:10.1371/journal.pone.0097149)
Supplement: Table S3 — ICD-10 pneumonia code recorded among 751 patients with pneumonia in new user cohort. 1. Patients may have multiple recordings of pneumonia codes. 2. HES ICD-10 codes recorded. *Descriptions were taken directly from the ICD-10. (DOCX) [file pone.0097149.s003.docx]

**Table S3**. ICD-10 pneumonia code recorded among 751 patients with pneumonia in new user cohort

| **ICD-10 diagnosis code** | **Description*** | **N** |
| --- | --- | --- |
| J18.1 | Lobar pneumonia, unspecified | 288 |
| J18.9 | Pneumonia, unspecified | 221 |
| J18.0 | Bronchopneumonia, unspecified | 85 |
| J69.0 | Pneumonitis due to food and vomit | 44 |
| J13 | Pneumonia due to Streptococcus pneumoniae | 7 |
| J14 | Pneumonia due to Haemophilus influenzae | 7 |
| J15.4 | Pneumonia due to other streptococci | 4 |
| J15.2 | Pneumonia due to staphylococcus | 3 |
| A16.9 | Resp TB unspec without mention of bact or hist confirm | 2 |
| J15.1 | Pneumonia due to Pseudomonas | 2 |
| J15.0 | Pneumonia due to Klebsiella pneumoniae | 2 |
| J85.2 | Abscess of lung without pneumonia | 2 |
| J15.9 | Bacterial pneumonia, unspecified | 1 |
| J15.6 | Pneumonia due to other aerobic Gram-negative bacteria | 1 |
| J11.0 | Influenza with pneumonia, virus not identified | 1 |
| J15.5 | Pneumonia due to Escherichia coli | 1 |
| B37.1 | Pulmonary candidiasis | 1 |
| J85.1 | Abscess of lung with pneumonia | 1 |
| A15.0 | TB lung confirm sputum microscopy with or without culture | 1 |
| A16.2 | TB lung without mention of bact or histological confirm | 1 |
| B01.2 | Varicella pneumonia | 1 |
| J17.2 | Pneumonia in mycoses | 1 |
| J16.8 | Pneumonia due to other specified infectious organisms | 1 |
| 1. Patients may have multiple recordings of pneumonia codes 2. HES ICD-10 codes recorded | | |

*Descriptions were taken directly from the ICD-10
