# Supplementary material for: Risk of Pneumonia with Inhaled Corticosteroid versus Long-Acting Bronchodilator Regimens in Chronic Obstructive Pulmonary Disease: A New-User Cohort Study
Source: PLoS One. 2014 May 30;9(5):e97149. doi: 10.1371/journal.pone.0097149 (PMC4039434; doi:10.1371/journal.pone.0097149)
Supplement: Table S4 — CPRD GOLD medcodes recorded among 751 patients with pneumonia. 1. Note that patients may have multiple recordings of pneumonia codes. 2. GPRD Medcodes recorded. *Descriptions were taken directly from the CPRD-COPD. (DOCX) [file pone.0097149.s004.docx]

Table S4. CPRD GOLD medcodes recorded among 751 patients with pneumonia

| **GPRD Medical Code (Events)** | **Description*** | **N** |
| --- | --- | --- |
| 572 | Pneumonia due to unspecified organism | 63 |
| 886 | Bronchopneumonia due to unspecified organism | 35 |
| 6094 | Pneumonia or influenza NOS | 21 |
| 1849 | Lobar (pneumococcal) pneumonia | 12 |
| 3683 | Basal pneumonia due to unspecified organism | 5 |
| 10086 | Pneumonia and influenza | 5 |
| 16287 | Chest infection - unspecified bronchopneumonia | 4 |
| 5202 | Viral pneumonia | 4 |
| 9639 | Lobar pneumonia due to unspecified organism | 4 |
| 14976 | Viral pneumonia NOS | 2 |
| 22795 | Chest infection - other bacterial pneumonia | 1 |
| 19400 | Chest infection - pneumonia due to unspecified organism | 1 |
| 13563 | Other aspiration pneumonia as a complication of care | 1 |
| 635 | Pulmonary tuberculosis | 1 |
| 38110 | Pulmonary tuberculosis NOS | 1 |
| 7133 | Respiratory TB not confirmed bact or histologically | 1 |
| 25694 | Pneumonia due to other specified organisms | 1 |
| 1. Note that patients may have multiple recordings of pneumonia codes 2. GPRD Medcodes recorded | | |

*Descriptions were taken directly from the CPRD-COPD
