# Supplementary material for: Risk of Pneumonia with Inhaled Corticosteroid versus Long-Acting Bronchodilator Regimens in Chronic Obstructive Pulmonary Disease: A New-User Cohort Study
Source: PLoS One. 2014 May 30;9(5):e97149. doi: 10.1371/journal.pone.0097149 (PMC4039434; doi:10.1371/journal.pone.0097149)
Supplement: Table S5 — Incidence of first pneumonia per 1000 person years for the final analysis cohort before and after propensity score balancing. IR: Incidence rate; PY: person years; Y: years. Severe pneumonia- pneumonia episode due to hospitalization or death during the pneumonia episode not censoring for prior non-severe pneumonia episode(s). (DOCX) [file pone.0097149.s005.docx]

**Table S5.** Incidence of first pneumonia per 1000 person years for the final analysis cohort before and after propensity score balancing

| **Variable** | **Before propensity score balancing** | | | | | | **After propensity score balancing**  **(matched cohorts)** | | | | | |
| --- | --- | --- | --- | --- | --- | --- | --- | --- | --- | --- | --- | --- |
|  | **ICS-containing**  **medications** | | | **LABD medications** | | | **ICS-containing medications** | | | **LABD medications** | | |
|  | **N=11,555** | | | **N=6,492** | | | **N=6,201** | | | **N=6,201** | | |
|  | **n** | **PY** | **IR per 1000 PY** | **n** | **PY** | **IR per 1000 PY** | **n** | **PY** | **IR per 1000 PY** | **n** | **PY** | **IR per 1000 PY** |
| **Any pneumonia**  **(secondary endpoint)** | 545 | 11,180.1 | 48.7 | 157 | 5,079.7 | 30.9 | 286 | 5,690.5 | 50.3 | 150 | 4,901.4 | 30.6 |
| Male | 346 | 6,062.3 | 57.1 | 86 | 2,872.2 | 29.9 | 193 | 3,367.9 | 57.3 | 83 | 2,758.7 | 30.1 |
| Female | 199 | 5,117.7 | 38.9 | 71 | 2,207.5 | 32.2 | 93 | 2,322.6 | 40.0 | 67 | 2,142.7 | 31.3 |
| Age at cohort entry date, y |  | | |  | | |  | | |  | | |
| 45–64 | 76 | 3,811.2 | 19.9 | 20 | 1,452.6 | 13.8 | 40 | 1,802.1 | 22.2 | 19 | 1,428.5 | 13.3 |
| 65–79 | 276 | 5,556.4 | 49.7 | 85 | 2,719.1 | 31.3 | 148 | 2,891.9 | 51.2 | 84 | 2,623.6 | 32.0 |
| ≥80 | 193 | 1,812.5 | 106.5 | 52 | 908.0 | 57.3 | 98 | 996.6 | 98.3 | 47 | 849.3 | 55.3 |
| **Severe pneumonia**  **(primary endpoint)** | 508 | 11,180.1 | 45.4 | 147 | 5,079.7 | 28.9 | 272 | 5,690.5 | 47.8 | 140 | 4,901.4 | 28.6 |
| Male | 316 | 6,062.3 | 52.1 | 82 | 2,872.2 | 28.5 | 180 | 3,367.9 | 53.4 | 79 | 2,758.7 | 28.6 |
| Female | 192 | 5,117.7 | 37.5 | 65 | 2,207.5 | 29.4 | 92 | 2,322.6 | 39.6 | 61 | 2,142.7 | 28.5 |
| Age at cohort entry date, y |  | | |  | | |  | | |  | | |
| 45–64 | 67 | 3,811.2 | 17.6 | 19 | 1,452.6 | 13.1 | 37 | 1,802.1 | 20.5 | 18 | 1,428.5 | 12.6 |
| 65–79 | 256 | 5,556.4 | 46.1 | 79 | 2,719.1 | 29.1 | 141 | 2,891.9 | 48.8 | 78 | 2,623.6 | 29.7 |
| ≥80 | 185 | 1,812.5 | 102.1 | 49 | 908.0 | 54.0 | 94 | 996.6 | 94.3 | 44 | 849.3 | 51.8 |
| **Hospitalized pneumonia** | 319 | 11,314.2 | 28.2 | 90 | 5,100.0 | 17.6 | 170 | 5,748.0 | 29.6 | 87 | 4,920.6 | 17.7 |
| Male | 197 | 6,156.2 | 32.0 | 51 | 2,880.9 | 17.7 | 111 | 3,410.6 | 32.5 | 50 | 2,766.5 | 18.1 |
| Female | 122 | 5,157.9 | 23.7 | 39 | 2,219.1 | 17.6 | 59 | 2,337.4 | 25.2 | 37 | 2,154.1 | 17.2 |
| Age at cohort entry date, y |  |  |  |  |  |  |  |  |  |  |  |  |
| 45–64 | 39 | 3,842.4 | 10.1 | 11 | 1,457.5 | 7.5 | 17 | 1,807.9 | 9.4 | 11 | 1,432.8 | 7.7 |
| 65–79 | 166 | 5,631.7 | 29.5 | 45 | 2,731.7 | 16.5 | 94 | 2,922.1 | 32.2 | 44 | 2,636.3 | 16.7 |
| ≥80 | 114 | 1,840.1 | 62.0 | 34 | 910.7 | 37.3 | 59 | 1,018.0 | 58.0 | 32 | 851.5 | 37.6 |
| **Hospitalized with pneumonia on the first episode** | 252 | 11,314.2 | 22.3 | 70 | 5,100.0 | 13.7 | 135 | 5,748.0 | 23.5 | 69 | 4,920.6 | 14.0 |
| Male | 155 | 6,156.2 | 25.2 | 40 | 2,880.9 | 13.9 | 88 | 3,410.6 | 25.8 | 40 | 2,766.5 | 14.5 |
| Female | 97 | 5,157.9 | 18.8 | 30 | 2,219.1 | 13.5 | 47 | 2,337.4 | 20.1 | 29 | 2,154.1 | 13.5 |
| Age at cohort entry date, y |  |  |  |  |  |  |  |  |  |  |  |  |
| 45–64 | 34 | 3,842.4 | 8.8 | 8 | 1,457.5 | 5.5 | 15 | 1,807.9 | 8.3 | 8 | 1,432.8 | 5.6 |
| 65–79 | 127 | 5,631.7 | 22.6 | 35 | 2,731.7 | 12.8 | 72 | 2,922.1 | 24.6 | 35 | 2,636.3 | 13.3 |
| ≥80 | 91 | 1,840.1 | 49.5 | 27 | 910.7 | 29.6 | 48 | 1,018.0 | 47.1 | 26 | 851.5 | 30.5 |
| IR: Incidence rate; PY: person years; Y: years  Severe pneumonia- pneumonia episode due to hospitalization or death during the pneumonia episode not censoring for prior non-severe pneumonia episode(s) | | | | | | | | | | | | |
