# Supplementary material for: Risk of Pneumonia with Inhaled Corticosteroid versus Long-Acting Bronchodilator Regimens in Chronic Obstructive Pulmonary Disease: A New-User Cohort Study
Source: PLoS One. 2014 May 30;9(5):e97149. doi: 10.1371/journal.pone.0097149 (PMC4039434; doi:10.1371/journal.pone.0097149)
Supplement: Table S6 — Hazard ratio and 95% CI of the association between first pneumonia and initiating medication use: applying sensitive to specific pneumonia definitions. CI: confidence interval; HR: hazard ratio; ICS: inhaled corticosteroids; LABD: long-acting bronchodilator. 1. Patients with a severe pneumonia episode due to a HES episode (hospitalization) with pneumonia as a primary diagnosis during an episode of care within the HES episode and not censored for other reasons during the severe pneumonia episode before the primary diagnosis. 2. Patients with a severe pneumonia episode due to hospitalization or death during the pneumonia episode not censoring for prior non-severe pneumonia episode(s). 3. Inverse probability of treatment weights method. (DOCX) [file pone.0097149.s006.docx]

**Table S6.** Hazard ratio and 95% CI of the association between first pneumonia and initiating medication use: applying sensitive to specific pneumonia definitions

| **Endpoint** | **ICS-containing new users** | **LABD**  **new users** | **Pneumonia events** | | **Crude** | **IPTW^3^** |
| --- | --- | --- | --- | --- | --- | --- |
|  | **N** | **N** | **ICS** | **LABD** | **HR (95% CI)** | **HR (95% CI)** |
| **Primary Model** | | | | | | |
| Any pneumonia (secondary endpoint) | 11,555 | 6,492 | 545 | 157 | 1.61 (1.34–1.92) | **1.49 (1.22–1.83)** |
| Severe pneumonia^2^ (primary endpoint) | 11,555 | 6,492 | 513 | 147 | 1.61 (1.34–1.94) | **1.57 (1.28–1.92)** |
| Hospitalized pneumonia^1^ | 11,555 | 6,492 | 319 | 90 | 1.62 (1.28–2.05) | **1.52 (1.16–1.98)** |
| Hospitalized with pneumonia on the first episode | 11,555 | 6,492 | 252 | 70 | 1.66 (1.27–2.17) | **1.55 (1.14–2.10)** |
| **New users with events ≥30 days following exposure** | | | | | | |
| Any pneumonia (secondary endpoint) | 11,333 | 5,954 | 472 | 142 | 1.50 (1.24–1.81) | **1.39 (1.12–1.72)** |
| Severe pneumonia^2^ (primary endpoint) | 11,333 | 5,954 | 444 | 133 | 1.50 (1.23–1.82) | **1.46 (1.17–1.82)** |
| Hospitalized pneumonia^1^ | 11,333 | 5,954 | 278 | 84 | 1.48 (1.16–1.89) | **1.41 (1.07–1.86)** |
| Hospitalized with pneumonia on the first episode | 11,333 | 5,954 | 220 | 67 | 1.48 (1.12–1.95) | **1.40 (1.02–1.92)** |
| **Persistent New Use (≥6 mo)** | | | | | | |
| Any pneumonia (secondary endpoint) | 5,549 | 3,847 | 386 | 121 | 1.46 (1.18–1.79) | **1.19 (0.93–1.52)** |
| Severe pneumonia^2^ (primary endpoint) | 5,549 | 3,847 | 360 | 115 | 1.42 (1.15–1.75) | **1.22 (0.97–1.55)** |
| Hospitalized pneumonia^1^ | 5,549 | 3,847 | 237 | 74 | 1.45 (1.12–1.89) | **1.24 (0.93–1.65)** |
| Hospitalized with pneumonia on the first episode | 5,549 | 3,847 | 186 | 57 | 1.49 (1.11–2.02) | **1.23 (0.89–1.72)** |
| **By ICS Dose** | | | | | | |
| Any pneumonia (secondary endpoint) | | | | | | |
| Low | 4,834 | 6,492 | 187 | 157 | 1.43 (1.15–1.77) | **1.37 (1.07–1.76)** |
| Medium | 4,545 | 6,492 | 205 | 157 | 1.49 (1.21–1.84) | **1.49 (1.19–1.86)** |
| High | 2,176 | 6,492 | 153 | 157 | 2.11 (1.68–2.65) | **1.86 (1.47–2.35)** |
| Severe pneumonia^2^ (primary endpoint) | | | | | | |
| Low | 4,834 | 6,492 | 176 | 147 | 1.43 (1.15–1.78) | **1.42 (1.10–1.83)** |
| Medium | 4,545 | 6,492 | 188 | 147 | 1.45 (1.17–1.80) | **1.49 (1.18–1.88)** |
| High | 2,176 | 6,492 | 149 | 147 | 2.20 (1.74–2.78) | **1.97 (1.55–2.49)** |
| Hospitalized pneumonia^1^ | | | | | | |
| Low | 4,834 | 6,492 | 99 | 90 | 1.30 (0.98–1.73) | **1.25 (0.91–1.72)** |
| Medium | 4,545 | 6,492 | 121 | 90 | 1.52 (1.15–2.00) | **1.50 (1.11–2.02)** |
| High | 2,176 | 6,492 | 99 | 90 | 2.35 (1.75–3.14) | **2.17 (1.61–2.93)** |
| Hospitalized with pneumonia on the first episode | | | | | | |
| Low | 4,834 | 6,492 | 77 | 70 | 1.33 (0.96–1.85) | **1.21 (0.84–1.72)** |
| Medium | 4,545 | 6,492 | 97 | 70 | 1.56 (1.14–2.12) | **1.57 (1.13–2.20)** |
| High | 2,176 | 6,492 | 78 | 70 | 2.39 (1.72–3.33) | **2.22 (1.58–3.12)** |
| **Original time period (2005**–**2010)** | | | | | | |
| Any pneumonia (secondary endpoint) | 6,937 | 5,000 | 326 | 114 | 1.66 (1.34–2.06) | **1.63 (1.31–2.04)** |
| Severe pneumonia^2^ (primary endpoint) | 6,937 | 5,000 | 307 | 109 | 1.63 (1.31–2.03) | **1.61 (1.29–2.03)** |
| Hospitalized pneumonia^1^ | 6,937 | 5,000 | 207 | 67 | 1.76 (1.33–2.33) | **1.70 (1.27–2.27)** |
| Hospitalized with pneumonia on the first episode | 6,937 | 5,000 | 155 | 53 | 1.69 (1.23–2.32) | **1.62 (1.16–2.25)** |
| CI: confidence interval; HR: hazard ratio; ICS: inhaled corticosteroids; LABD: long-acting bronchodilator   1. Patients with a severe pneumonia episode due to a HES episode (hospitalization) with pneumonia as a primary diagnosis during an episode of care within the HES episode and not censored for other reasons during the severe pneumonia episode before the primary diagnosis 2. Patients with a severe pneumonia episode due to hospitalization or death during the pneumonia episode not censoring for prior non-severe pneumonia episode(s) 3. Inverse probability of treatment weights method | | | | | | |
