# Supplementary material for: Risk of Pneumonia with Inhaled Corticosteroid versus Long-Acting Bronchodilator Regimens in Chronic Obstructive Pulmonary Disease: A New-User Cohort Study
Source: PLoS One. 2014 May 30;9(5):e97149. doi: 10.1371/journal.pone.0097149 (PMC4039434; doi:10.1371/journal.pone.0097149)
Supplement: Table S7 — Summary of studies investigating the association between pneumonia and use or non-use of ICS. ACE: angiotensin-converting-enzyme; CAP: community-acquired pneumonia; CHF: congestive heart failure; ER: emergency room; FEV1: forced expiratory volume in 1s; GERD: gastroesophageal reflux disease; GP: general practitioner; HR: hazard ratio; ICS: inhaled corticosteroid; LABA: long-acting β2-agonist; LABD: long-acting bronchodilator; MI: myocardial infarction; NSAIDS: non-steroidal anti-inflammatory drug; OCS = oral corticosteroid; OR: odds ratio; RR: rate ratio; Rx: prescription; SABD: short-acting bronchodilator; VA: Veterans Affairs. (DOCX) [file pone.0097149.s007.docx]

**Table S7.** Summary of studies investigating the association between pneumonia and use or non-use of ICS

| **Author** | **Endpoint** | **Dose-Response** | **Type of ICS Use** | **Method** | **Outcome(s)** | **Other Notes** |  |
| --- | --- | --- | --- | --- | --- | --- | --- |
| **Ernst, 2007 [1]** | Hospitalization for pneumonia (primary diagnosis)  Hospitalization and mortality within 30 days | Observed | Current  (within 60 days)  Current by Dose  Past ICS Use | - Nested Case-Control  - 1998-2003; ≥66 years old  - 4 controls matched to 1 case on age and time  - Cohort with 3 or more prescriptions for any form of β-agonist, theophylline, ipratropium bromide, sodium cromoglycate, nedocromil, or ketotifen in 1 year; cohort entry on date of 3^rd^ prescription  - **Adjustment for confounding:**  Adjustment (logistic regression) for markers of disease severity - number of dispensed respiratory medications (β-agonists, ipratropium bromide, theophylline, and cromolyns), OCS, antibiotics, and COPD hospitalization (in the past year)  Stratification by OCS or COPD hospitalization in the past year | **Hospitalization:**  RR=1.70 (1.63-1.77)  Current high:  RR=2.25 (2.07-2.44)  Current medium:  RR=1.63 (1.55-1.71)  Current low:  RR=1.50 (1.38-1.62)  **Hospitalization and death w/in 30 days:**  RR=1.53 (1.30-1.80) | - Comorbidities associated with risk of pneumonia based on prescriptions associated with the treatment of disorders dispensed in the past year  -No measures of lung function; Patients with asthma were excluded  -Equivalent doses of ICS based on relative topical potency into fluticasone equivalents: beclomethasone 100 µg= budesonide 80 µg= triamcinolone 200 µg= fluticasone 50 µg= flunisolide 200 µg 🡪 high dose (fluticasone, 1,000 µg/d or more), moderate dose (fluticasone, 500–999 µg/d), and low dose (fluticasone, less than 500 µg/d) |  |
| **Mapel, 2010 [2]** | Any pneumonia  (outpatient, emergency room, or inpatient) | Not done | Current continuous treatment of 90, 180, and 365 days prior to case  (30 days in script +45 day gap allowed) | - Nested Case-Control  - 2001-2003; US Managed care  - 4 controls matched to each case on age, sex and date of COPD diagnosis  - **Adjustment for confounding:**  Adjustment (logistic regression) for disease severity- COPD hospital, ER visits and outpatient visits, OCS (in the prior 12 months) | **Any pneumonia**  90-days current use  ICS alone  OR=1.29 (0.96-1.73) Fluticasone propionate/ salmeterol combination  OR=1.03 (0.74-1.42)  ICS with LABA  OR=0.58 (0.30-1.12)  *compared with SABD treated | - Covariates also included age, sex, prior pneumonia, asthma diagnosis, and Charlson score in the past year  - Asthma patients included  -No lung function data; no smoking data  - ER and outpatient were required to have a chest x-ray |  |
| **Joo, 2010 [3]** | Hospitalization for pneumonia (primary diagnosis)  Mortality within 30 days of hospitalization | Analysis conducted, but dose-response was “not noted” | Current (within 90 days)  Current by dose  Current use + recent OCS use (31-180 days)  Past ICS use (91-365 days before)  Dose and Current/  Past use | - Nested Case-Control  - 1998-2003; VA patients (≥65 years old)  - Up to 10 controls matched to each case on age, sex, and cohort entry date  - **Adjustment for confounding:**  Newly diagnosed COPD (within the past year) in order to eliminate cumulative corticosteroid exposure and those with severe COPD  Adjustment (logistic regression) for disease severity- dispensing of respiratory medications, OCS, primary care and ER visits, COPD exacerbations (in the year before event date) | **Hospitalization:**  OR=1.38 (1.31-1.45)  Current high:  OR=1.30 (1.21-1.41)  Current medium:  OR=1.28 (1.18-1.39)  Current low:  OR=1.51 (1.41-1.62)  **Mortality within 30 days of hospitalization**  Current use:  13.3% died  Without current use: 17.2% died | - Covariates also included age, comorbidities, and medication classes associated with pneumonia (including benzodiazepines). Comorbidities assessed through diagnostic codes and medication dispensing  -No lung function data; No smoking history data; Concomitant asthma patients excluded  -Cases appeared to have more severe COPD compared with controls  Sensitivity analysis excluded those with chronic OCS use  - Doses were converted to beclomethasone equivalents: low (≤1000 µg per day), moderate (>1000 µg -2000 µg per day), and high (>2000 µg per day) doses |  |
| **Thornton Snider,**  **2012 [4]** | Any pneumonia  (hospital or outpatient visit for pneumonia as the primary diagnosis occurring at least 1 year after cohort entry) | Observed | Any  (past year)  Current (within 90 days)  Current by dose  Past ICS use (91-365 days before)  Dose and Current/  Past use | - Nested Case-Control  - 2007-2011; Medicare population with Medicare part D (≥65 year old)  - 2 to 3 controls matched to each pneumonia case on cohort entry, age, and sex  - **Adjustment for confounding:**  Adjustment (logistic regression) for disease severity- respiratory medication use, antibiotics, OCS, primary care and ER visits, prior history of COPD, COPD exacerbations (in the past year)  Sensitivity analyses: newly diagnosed COPD only; only hospitalization (no outpatient) for pneumonia; differing lengths and dose of ICS; PS for any, current, and past ICS use | **Any pneumonia**  Any ICS use:  OR=1.11 (1.05–1.18)  Current use:  OR=1.26 (1.16-1.36)  Current high:  OR=1.55(1.25-1.92)  Current medium:  OR=1.39 (1.25-1.55)  Current low:  OR=1.11 (1.00-1.23)  **Results of sensitivity analyses were similar** | -Covariates also identified in the past year included age, sex, comorbid conditions, and pneumococcal vaccine  - Asthma patients excluded; Patients with a prior COPD diagnosis were flagged and excluded from sensitivity analyses  - No smoking data; no lung function data  - Doses were converted to fluticasone equivalents: low dose (<500 µg/day for current use, <406 µg/day for past use only); medium dose (<833 µg/day for current use,<670 µg/day for past use only, and greater than low dose); high dose (≥833 µg/day for current use, ≥670 µg/day for past use only, and greater than low dose) |  |
| **Yawn, 2013 [5]** | Any pneumonia (outpatient, ER, or inpatient) | Observed | Any ICS exposure  ICS by dose* | - Retrospective cohort  - 2006-2010; Marketscan Commercial claims and CMS (≥45 years old)  **- Adjustment for confounding:**  Newly diagnosed COPD (within the past year) in order to eliminate cumulative corticosteroid exposure and those with severe COPD  Patients were excluded if they had ICS use in the 12-months before COPD diagnosis | **Any pneumonia**  Any exposure  HR=1.51 (1.42-1.61)  High dose:  HR=2.57 (1.98-3.33)  Medium dose:  HR=1.69 (1.52-1.88)  Low dose:  HR=1.38 (1.27-1.49)  **Sensitivity analysis controlling for OCS use found similar dose response magnitudes** | - Covariates (Cox proportional hazard) included age, sex, region, insurance type, COPD diagnosis year, respiratory medications, comorbidities, and all cause ER visits and hospitalizations (12 months prior to COPD diagnosis)  - Asthma patients excluded; Patients using OCS or who had a history of pneumonia during the year before COPD diagnosis were also excluded  - No lung function data; no smoking data  - “details on severity of disease were not available to us”  - Doses were converted to fluticasone equivalents: low (<500 μg/day), moderate (500 μg/day to <1000 μg/day) and high (≥1000 μg/day) dose |  |
| **Suissa, 2013 [6]** | Serious pneumonia  (hospitalization for pneumonia as the reason for admission/ primary diagnosis or death from pneumonia) | Observed | Current (within 60 days)  Past use  (61-365 days before)  Current use by dose  Current use by ICS (fluticasone, budesonide, other ICS) and dose | - Nested case-control among new users  - 1990-2007 (new users identified between 1990-2005); Computerized databases of health insurance claims, deaths, and prescription medications in Quebec, Canada; ≥55 years old  -Cohort with 3 or more prescriptions for any β-agonist, theophylline, ipratropium, or tiotropium bromide in 1 year; cohort entry on date of 3^rd^ prescription  -New users with COPD had no respiratory medication use (any β-agonist, theophylline, ipratropium, tiotropium bromide, or ICS) in the 2 years before the 1^st^ of the 3 prescriptions defining cohort entry  - 10 controls matched to 1 case on age and cohort entry  **- Adjustment for confounding:**  New users of respiratory medication  Adjustment (logistic regression) for severity of respiratory disease- respiratory medication prescriptions, OCS prescriptions, and hospitalization for COPD in the year prior to index  Sensitivity analysis stratifying by prior COPD hospitalization and ICS type | **Serious Pneumonia**  Current use:  RR=1.69 (1.63-1.75)  Current high:  RR=1.86 (1.77-1.94)  Current medium:  RR=1.66 (1.59-1.74)  Current low:  RR=1.24 (1.13-1.36)  Current fluticasone:  RR=2.01 (1.93-2.10)  Current budesonide:  RR=1.17 (1.09-1.26)  Current Other ICS (primarily beclomethasone):  RR=1.41 (1.33-1.51) | - Covariates also identified in the past year included sex, age, and other conditions associated with risk of pneumonia (drugs for cardiac disease, central nervous system including benzodiazepines, osteoporosis, as well as antidepressive agents, narcotics, and NSAIDs) based on medication prescriptions  - Confounding variables, i.e. other conditions and severity of respiratory disease, based on prescriptions in the past year  - Asthma patients excluded  - No lung function data; no smoking data  - Chest x-rays were not required, but likely based on a primary inpatient diagnosis being required  - Dose equivalences used were beclomethasone 100 μg = budesonide 80 μg = triamcinolone 200 μg = fluticasone 50 μg = flunisolide 200 μg categorized as high (fluticasone ≥1000 μg/day), moderate (500–999 μg/day) and low (< 500 μg/day) based on fluticasone equivalents  - The prescription drugs database included outpatient prescription medications dispensed to all people aged 65 years or older, social welfare recipients and, since 1996, all other residents who opted to join the provincial drug plan, covering around half the population of Quebec.  - “Of most concern is the possibility that budesonide may have been preferentially prescribed to patients with a lower risk of pneumonia, such as those with asthma or less severe COPD.” |  |
| *Modeled as time dependent, dosage defined on a daily basis, and so one patient may contribute to different levels of ICS use or no use depending on daily exposure | | | | | | | |

ACE: angiotensin-converting-enzyme; CAP: community-acquired pneumonia; CHF: congestive heart failure; ER: emergency room; FEV_1_: forced expiratory volume in 1s; GERD: gastroesophageal reflux disease; GP: general practitioner; HR: hazard ratio; ICS: inhaled corticosteroid; LABA: long-acting β_2_-agonist; LABD: long-acting bronchodilator; MI: myocardial infarction; NSAIDS: non-steroidal anti-inflammatory drug; OCS = oral corticosteroid; OR: odds ratio; RR: rate ratio; Rx: prescription; SABD: short-acting bronchodilator; VA: Veterans Affairs.

**References**

1. Ernst P, Gonzalez AV, Brassard P, Suissa S (2007) Inhaled corticosteroid use in chronic obstructive pulmonary disease and the risk of hospitalization for pneumonia. Am J Respir Crit Care Med 176: 162–166.
2. Mapel D, Schum M, Yood M, Brown J, Miller D, Davis K (2010) Pneumonia among COPD patients using inhaled corticosteroids and long-acting bronchodilators. Prim Care Respir J 19: 109–117.
3. Joo MJ, Au DH, Fitzgibbon ML, Lee TA (2010) Inhaled corticosteroids and risk of pneumonia in newly diagnosed COPD. Respir Med 104: 246–252.
4. Thornton Snider J, Luna Y, Wong KS, Zhang J, Chen SS, Gless PJ, et al. (2012) Inhaled corticosteroids and the risk of pneumonia in Medicare patients with COPD. Curr Med Res Opin 28: 1959–1967.
5. Yawn BP, Li Y, Tian H, Zhang J, Arcona S, Kahler KH (2013) Inhaled corticosteroid use in patients with chronic obstructive pulmonary disease and the risk of pneumonia: a retrospective claims data analysis. Int J Chron Obstruct Pulmon Dis 8: 295–304.
6. Suissa S, Patenaude V, Lapi F, Ernst P (2013) Inhaled corticosteroids in COPD and the risk of serious pneumonia. Thorax 68: 1029–1036.
